# Supplementary material for: Living with a diagnosis of Placenta Accreta Spectrum: Mothers’ and Fathers’ experience of the antenatal journey and the birth
Source: PLoS One. 2023 May 22;18(5):e0286082. doi: 10.1371/journal.pone.0286082 (PMC10202293; doi:10.1371/journal.pone.0286082)
Supplement: S1 File — (DOCX) [file pone.0286082.s001.docx]

**The subjective experience of suffering from Placenta Accreta Spectrum (PAS)**

1) How did you experience your pregnancy (before the diagnosis of PAS)?

2) What was it like for you to receive the diagnosis of PAS?

What were your thoughts and your feelings?

What were your anxiety/worries?

How did you cope with this diagnosis?

What kind of support systems were helpful?

3) What was your experience of the remainder of the pregnancy after having received the diagnosis of PAS?

What were your thoughts and your feelings?

What were your anxiety/worries?

How did you cope with this diagnosis?

What kind of support systems were helpful?

4) How would you describe your birth experience?

What were your thoughts and your feelings?

What were your anxiety/worries?

How did you cope?

What kind of support systems were helpful?

The interview guide was modified where partners were interviewed to allow emergence of a systemic perspective.
