# Supplementary material for: Living with a diagnosis of Placenta Accreta Spectrum: Mothers’ and Fathers’ experience of the antenatal journey and the birth
Source: PLoS One. 2023 May 22;18(5):e0286082. doi: 10.1371/journal.pone.0286082 (PMC10202293; doi:10.1371/journal.pone.0286082)
Supplement: Attachment — Submitted filename: Response to reviewers.docx [file pone.0286082.s002.docx]

Dear Prof Desseauve,

Thank you to the reviewers for their comments on our manuscript. Please see below a point-by-point response to their review.

The funding statement for this research is as follows:

*This research received funding from the National Maternity Hospital Foundation, a registered charity operating in Ireland (CHY 20389 registration number). The funders had no role in study design, data collection and analysis, decision to publish, or preparation of the manuscript.*

Kind regards,

Dr Helena Bartels

**Journal Requirements:**

**When submitting your revision, we need you to address these additional requirements.**

**1. Please ensure that your manuscript meets PLOS ONE's style requirements, including those for file naming. The PLOS ONE style templates can be found at** 
[**https://journals.plos.org/plosone/s/file?id=wjVg/PLOSOne_formatting_sample_main_body.pdf**](https://journals.plos.org/plosone/s/file?id=wjVg/PLOSOne_formatting_sample_main_body.pdf) **and** 
[**https://journals.plos.org/plosone/s/file?id=ba62/PLOSOne_formatting_sample_title_authors_affiliations.pdf**](https://journals.plos.org/plosone/s/file?id=ba62/PLOSOne_formatting_sample_title_authors_affiliations.pdf)

**2. We note that the grant information you provided in the ‘Funding Information’ and ‘Financial Disclosure’ sections do not match.**

**When you resubmit, please ensure that you provide the correct grant numbers for the awards you received for your study in the ‘Funding Information’ section.**

Thank you for this comment. There is no grant number which goes along with this funding, however we have ensured the wording is the same for the cover letter and metadata at submission. (See below, point 3)

**3. Thank you for stating the following in the Acknowledgments Section of your manuscript:**

**This research was funded by the National Maternity Hospital Foundation.**

**However, funding information should not appear in the Acknowledgments section or other areas of your manuscript. We will only publish funding information present in the Funding Statement section of the online submission form. 
Please remove any funding-related text from the manuscript and let us know how you would like to update your Funding Statement. Currently, your Funding Statement reads as follows:**

**This research received funding from the National Maternity Hospital Foundation, a registered charity operating in Ireland (CHY 20389 registration number). The funders had no role in study design, data collection and analysis, decision to publish, or preparation of the manuscript.**

**Please include your amended statements within your cover letter; we will change the online submission form on your behalf.**

Thank you for this comment. We have removed the fundings statement from the acknowledgements section, and from the end of the manuscript as requested. We confirm the funding statement as outlined above is correct. We have included this statement in the cover letter.

**4. Please note that in order to use the direct billing option the corresponding author must be affiliated with the chosen institute. Please either amend your manuscript to change the affiliation or corresponding author, or email us at** [**plosone@plos.org**](mailto:plosone@plos.org) **with a request to remove this option.**

Thank you for this comment. I have emailed the address as instructed. We would like to proceed with payment by bank transfer or payment link.

**5. Your ethics statement should only appear in the Methods section of your manuscript. If your ethics statement is written in any section besides the Methods, please delete it from any other section.**

The ethics statement is in the methods section, and has now been removed from the end of the manuscript.

**6. Please include captions for your Supporting Information files at the end of your manuscript, and update any in-text citations to match accordingly. Please see our Supporting Information guidelines for more information:** [**http://journals.plos.org/plosone/s/supporting-information**](http://journals.plos.org/plosone/s/supporting-information)**.**

Thank you we have now included a reference to supporting information S1 on line 168 in the text, and included a reference to this at the end of the manuscript.

**7. Please review your reference list to ensure that it is complete and correct. If you have cited papers that have been retracted, please include the rationale for doing so in the manuscript text, or remove these references and replace them with relevant current references. Any changes to the reference list should be mentioned in the rebuttal letter that accompanies your revised manuscript. If you need to cite a retracted article, indicate the article’s retracted status in the References list and also include a citation and full reference for the retraction notice.**

**Review Comments to the Author**

**Reviewer #1: this is a very interesting paper and an original way to address psychological impact of PAS while including the partner that usually is under stress and often overlooked. Many couples have dysfunction in their relationship due to distress experienced not only by patients but by other members of the close family (husband and children)
this paper is very well written, is descriptive and is well discussed.
It is very difficult to produce such a kind of paper especially when it is descriptive.**

Thank you for your helpful and positive comments on our manuscript.

**Some minor errors as on line 137 the age should be >= 18**

Thank you for pointing this out, we have updated.  **I would ask about if there was a difference between women interviewed lately (around 5 years after) and those who experienced PAS delivery within few months. I think that time can erase or mask some bad memories and render it more acceptable.**

Thank you for this comment. The median (IQR) at the time of interview since the PAS pregnancy was 18 months (12.5–21.5). We did not include anyone who had a PAS pregnancy more than 5 years ago (inclusion criteria within 5 years). Most participants were between 1.5-2.5 years after the birth. We did not observe any differences in the themes between those who were more recent compared to further removed from the birth. Traumatic experiences are by their nature particularly well ingrained in the memory. We did not ask participants to remember detail in terms of dates, times, people etc. It was very much focussed on their experience of PAS.

We have added to the paper on line 187: “The median (IQR) at the time of interview since the PAS pregnancy was 18 months (12.5–21.5). We did not ask participants to remember detail in terms of dates, times, people etc. The interview was very much focussed on their experience of PAS. Unsurprisingly, because of the traumatic experience of PAS, each participant indicated at interview they could recall vividly key aspects of their experience.”.

**Moreover, it is interesting for differentiate between those interviewed with their husband and those without their husband is there any difference in the reaction between the group of five without husband interviewed and those interviewed separately from their partner?**

Thank you for this interesting comment and it is something we considered in the study design and analysis. Many of the women who were interviewed alone choose to do so for very pragmatic reasons, such as childcare arrangements. We did not observe a difference in the themes or interviews between those interviewed together or separately. We did find that some women who were interviewed alone said their partner would not be willing to partake, however the experiences of these women did not appear to be different from those who were interviewed with their partner. Of note, all women who were interviewed alone were still in the relationship and there were no single or separated women interviewed. Our impression is there was no difference, but we did not compare these groups. We have now included this as a limitation on line 479 “Furthermore, it is possible where women were interviewed with their partner present that this influenced the nuances of the topics discussed. However, in this sample, differences in the data were not apparent based on whether the couples were interviewed together or separately.”

**This paper underlines the importance of management since the beginning by a multidisciplinary team specialized in PAS. it learns us what is lacking in the management of these patients. Sometimes small and insignificant things for us is major and paramount for patient ( experiencing cold, freezing... this is a period the patient thinks she has to go through because she wants to finish with this delivery...but could be avoided by the PAS team with some attention). Publication of this paper is an important testimony for those dealing with PAS

I congratulate the authors for their incredible work and hope seing it published as soon as possible**

Thank you for your supportive and positive comments on our manuscript.

**Reviewer #2: Thank you to the editor for giving me the opportunity to review this manuscript which presents the results of a qualitative study on the experiences of couples with pregnancies marked by placenta accretion.
This is an ancillary analysis of the same material presented in another publication (**[**https://doi.org/10.1186/s12884-022-04726-8**](https://doi.org/10.1186/s12884-022-04726-8)**). This material consists of 23 interviews with 29 participants. These participants were 17 women with placenta accreta and 12 partners.

The stated aim of this article is to assess the psychological impact of accretion, whereas the aim of their previous article was to describe the experiences of these women and their partners.
In my opinion, a qualitative study should avoid using the terms "impact assessment" but rather talk about identification and/or understanding.**

Thank you for this comment. We have now removed the word “impact” from the abstract and throughout the manuscript, and replaced with “consequences”.  **In the abstract, it seems to me that the authors used the term "Placenta Accreta Syndrome" instead of "Placenta Accreta Spectrum". Please confirm.**

Thank you for pointing this out. We have updated to the correct terminology of “Placenta Accreta Spectrum”, as used throughout the rest of the abstract and manuscript.

**Some errors in the referencing of studies, lines 408 and 473.**

Thank you for highlighting this, we have now updated these references.

**The introduction is clear and does not require any comment from me.

The method is rigorous. It might be interesting to clarify the context of care in Ireland, including how women should be informed of the risks. Indeed, in some countries women are systematically informed of all the risks involved, including the darker ones, with their prevalence, whereas in other countries the information is less structured and the numerical risks are not communicated to parents.**

Thank you for this comment. In Ireland, standard practice (1) would be to fully counsel women of serious risks involved in any medical or pregnancy condition with which they are diagnosed, including the risk of death. We have previously written on this area (2) and it would be considered standard for healthcare providers to fully disclose all risks which a ‘reasonable’ person would wish to know. However, we could not say for certain that each woman was told the risks in a similar manor, as the interviewed participants attended multiple different care providers. We have added a line in the limitations to address this also, line 490” For the latter, women in this site were fully informed of as to the risks of PAS including loss of life”

**Regarding the results, I think it is important to propose a coding tree (in a figure or a table) that is consistent with the aim of this article.

Here is the current structure:
1. Antenatal

1.1. Living with PAS
1.1.1. Lack of knowledge
1.1.2. Experience of care

1.2. Coping with uncertainty
1.2.1. Getting on with it
1.2.2. Emoional toll


2. Birth

2.1. A traumatic experience
2.1.1. Sayying goodbye
2.1.2. Experienceing trauma
2.1.3. Witnessing of trauma

2.2. Feeling safe in the hands of experts
2.2.1. Safety in expert team
2.2.2. Relief at surviving

I believe that the terms chosen to describe the themes could be more explicit to facilitate the link with the aim of the article.
For example, how does surbordinate themes "1.1.1. Lack of knowledge/information" is related to the stated aim line 109 of assessing the psychological impact of the PAS? On the other hand, the issue of lack of information seems to me to have already been published in the section "What needs to change" (**[**https://doi.org/10.1186/s12884-022-04726-8**](https://doi.org/10.1186/s12884-022-04726-8)**). The authors should consider renaming this theme "fear of the unknwon" for example. Another theme could be "the reassuring side of care planning". I therefore recommend that the authors revise the names "Lack of knowledge" and "Experience of care" to make them consistent with the aim of their analysis.**

Thank you for these insightful and interesting comments. We agree that the themes could be more descriptively named and we are grateful for your suggestions. In terms of “lack of knowledge”, we have added clarity by expanding the title to “lack of knowledge of PAS”. While we appreciate your comment on fear of the unknown, we feel this is addressed under the theme of “coping with uncertainty”, and under the “emotional toll” section. Whilst women who were booked with the MDT team had reassurances of care, this was not the case for women who did not receive this level of care in smaller regional units. Therefore “experiences of care” theme was titled as such to reflect the wide and varied experiences of care. However we appreciate the comment and have altered this to “experiences of varied approaches to care”.

We have also included a figure to clearly show the thematic map of superordinate, main and subthemes as suggested (named Figure 1). The coding has been updated as suggested starting with theme 1, then superordinate themes 1.1 etc. (Tables and manuscript updated).

**It would be interesting to know which themes had been anticipated (and were present in the interview grid) and which themes emerged from the data.**

Thank you for this comment. There was very limited literature available at the time of the study design and recruitment exploring this topic. Therefore, the authors did not anticipate any themes in particular, as the literature in this areas was largely unknown. An interpretive phenomenological analysis approach was taken to explore these unique and largely unknown experiences, to allow us to uncover new and unknown concepts.  **The saturation of themes is defined in the line 159 method but is not addressed in the results. Do the authors think they have reached saturation and can they specify this?**

Thank you for this comment. In a small and rare population, of course we can never be certain of reaching data saturation. However, we did not complete our data collection until no new themes were emerging in the sample to which we had access. Therefore, we have added this as further explanation to the methods on line 161: “Saturation of themes was defined when refinements did not add any new substantial themes. By this we mean we did not complete our data collection or analyses until no new themes were emerging in the sample to which we had access”.

**In the discussion section, it would be more explicit to use the words used in the objective line 109 to construct the first sentence of the discussion line 385.**

Thank you for this comment. We have now updated the first sentence of the introduction to reflect the language used in the objective line, line 385 now reads: “This study explored the psychological consequences of PAS on women and their support partners during pregnancy, up to and including the birth.”

**The rest of the discussion is clear and makes important points for clinicians as well.**

**It would be interesting to discuss the context of care in Ireland and data saturation in the strengths and weaknesses section.**

Thank you for this comment. We have added the following in relation to context of care in Ireland to the limitations: “When these participants were receiving care, some received specialist PAS MDT care, while others were cared for in a standard care setting. For the former, women in this site were fully informed of as to the risks of PAS including loss of life. As maternal outcomes are significantly improved when women are care for by a PAS MDT, a recently published clinical care guideline in Ireland has recommended a move to a national PAS MDT. ”

Data saturation was addressed on line 161 in methods.  **This article meets research standards and adds substantially to the existing literature. I therefore recommend its publication.**

**6. PLOS authors have the option to publish the peer review history of their article (**[**what does this mean?**](https://journals.plos.org/plosone/s/editorial-and-peer-review-process#loc-peer-review-history)**). If published, this will include your full peer review and any attached files.


If you choose “no”, your identity will remain anonymous but your review may still be made public.


Do you want your identity to be public for this peer review? For information about this choice, including consent withdrawal, please see our** [**Privacy Policy**](https://www.plos.org/privacy-policy)**.**

**Reviewer #1: Yes: David Atallah**

**Reviewer #2: Yes: Laurent GAUCHER**

**References**

1.	Guide to Professional Conduct and Ethics for Registered Medical Practitioners

(Amended). 2019. p. <https://www.medicalcouncil.ie/news-and-publications/reports/guide-to-professional-conduct-and-ethics-for-registered-medical-practitioners-amended-.pdf>.

2.	Bartels HC, Brennan DJ. Authors' response to letter to the editor: Quality of life after placenta accreta spectrum: Should women be informed of mortality risk? The Australian & New Zealand journal of obstetrics & gynaecology. 2021;61(4):E25.
